# Occupation, work-related contact and SARS-CoV-2 anti-nucleocapsid serological status: findings from the Virus Watch prospective cohort study

**DOI:** 10.1136/oemed-2021-107920

**Published:** 2022-04-21

**Authors:** Sarah Beale, Parth Patel, Alison Rodger, Isobel Braithwaite, Thomas Byrne, Wing Lam Erica Fong, Ellen Fragaszy, Cyril Geismar, Jana Kovar, Annalan Navaratnam, Vincent Nguyen, Madhumita Shrotri, Anna Aryee, Robert Aldridge, Andrew Hayward

**Affiliations:** 1 UCL Institute of Epidemiology and Health Care, University College London, London, UK; 2 UCL Institute of Health Informatics, University College London, London, UK; 3 Extreme Events and Health Protection Team, Centre for Radiation, Chemicals and Environmental Hazards, Public Health England, London, UK; 4 Department of Infectious Disease Epidemiology, London School of Hygiene and Tropical Medicine, London, UK

**Keywords:** COVID-19, Occupational Health, Epidemiology

## Abstract

**Objectives:**

Risk of SARS-CoV-2 infection varies across occupations; however, investigation into factors underlying differential risk is limited. We aimed to estimate the total effect of occupation on SARS-CoV-2 serological status, whether this is mediated by workplace close contact, and how exposure to poorly ventilated workplaces varied across occupations.

**Methods:**

We used data from a subcohort (n=3775) of adults in the UK-based Virus Watch cohort study who were tested for SARS-CoV-2 anti-nucleocapsid antibodies (indicating natural infection). We used logistic decomposition to investigate the relationship between occupation, contact and seropositivity, and logistic regression to investigate exposure to poorly ventilated workplaces.

**Results:**

Seropositivity was 17.1% among workers with daily close contact vs 10.0% for those with no work-related close contact. Compared with other professional occupations, healthcare, indoor trade/process/plant, leisure/personal service, and transport/mobile machine workers had elevated adjusted total odds of seropositivity (1.80 (1.03 to 3.14) − 2.46 (1.82 to 3.33)). Work-related contact accounted for a variable part of increased odds across occupations (1.04 (1.01 to 1.08) − 1.23 (1.09 to 1.40)). Occupations with raised odds of infection after accounting for work-related contact also had greater exposure to poorly ventilated workplaces.

**Conclusions:**

Work-related close contact appears to contribute to occupational variation in seropositivity. Reducing contact in workplaces is an important COVID-19 control measure.

**Key messages**

**What is already known about this subject?**

- SARS-CoV-2 infection risk appears to vary across occupational groups, with official statistics and research findings across a range of global regions demonstrating elevated risk for healthcare, social care, transport, and food service and other personal service workers. Elevated infection risk in these occupations was hypothesised to be influenced by in-person workplace attendance and/or contact with patients or the public across studies.

**What are the new findings?**

- We investigated the effect of occupation on natural infection-related seropositivity (up to June 2021) from a cohort in England and Wales, building on previous studies by controlling for a range of potential confounders and investigating the mediating effect of work-related contact. Workers in healthcare, indoor trade, process and plant, and leisure and personal service occupations had elevated total odds of seropositivity for SARS-CoV-2 natural infection. Work-related contact accounted for a variable proportion of the relationship between occupation and odds of seropositivity across occupational groups. Occupational groups in which a direct effect remained after adjustment for non-household contact reported greater odds of frequent exposure to poorly ventilated workplaces.

**Key messages**

**How might this impact on policy or clinical practice in the foreseeable future?**

- Current evidence suggests that work-related contact with non-household members is an important factor influencing differential risk of SARS-CoV-2 across occupations. Reducing frequency of work-related contact by supporting working from home where possible and implementing social distancing and other risk mitigation methods in workplaces is likely to influence work-related SARS-CoV-2 transmission. Further inquiry into the inter-relationship between work-related contact and other features of the workplace, including ventilation, is warranted to inform public health interventions and policy.

## Introduction

Occupation is a major determinant of health,^1^ and is hypothesised to drive exposure to SARS-CoV-2 during the current pandemic by influencing people’s ability to work from home, practice social distancing and work in well-ventilated conditions.^2–4^

Substantial occupational differences in severe illness and mortality have emerged worldwide,^5–9^ with official statistics in the UK, USA, Sweden and Brazil identifying excess risk for various occupational groups, including healthcare^3 9–11^ and care-related occupations,^3 6–9^ transportation workers,^3 6–10 12^ essential trades and process/plant occupations,^3 6–9 11^ personal service occupations^3 6–9 11^ and protective service workers^3 9 11^ compared with other occupations or the general population. These occupational groups are broadly patient or public facing and/or require work outside the home,^2–4^ potentially increasing SARS-CoV-2 exposure risk. Occupational differences in severe illness and COVID-19 mortality may be related to workplace exposure, but this cannot be easily inferred from such studies.

Emerging evidence suggests that infection risk varies substantially across occupations. Rates of antigen test positivity and nucleocapsid antibody seropositivity in healthcare workers—who are at high risk of contact with infectious individuals^3^ —usually exceed those observed in the general population but vary across study populations.^13–20^ However, estimates were largely drawn from the initial phase of the pandemic, when access to personal protective equipment (PPE) was variable and healthcare workers had greater access to SARS-CoV-2 testing than the general population.^2^ Early evidence from contact tracing^21^ and routine population testing^22^ including a range of occupations across several global regions suggested workplaces as a common plausible location of transmission, with cases higher among occupations involving public exposure, including transport, hospitality, cleaning and service occupations.

Studies investigating occupational differences in COVID-19 risk based on only the first pandemic wave, which comprises most of the existing literature, should be interpreted with caution due to changes in differential risk by occupation across pandemic waves. In the UK, employer-submitted reports of COVID-19 cases plausibly linked to the workplace^23 24^ primarily involved health and social care workers in the first pandemic wave compared with education and manufacturing workers in the second pandemic wave. Although reporting bias may have influenced these findings, patient-facing healthcare and care workers participating in a randomly sampled antigen testing study demonstrated higher rates of current infection than other workers in May 2020 but at no subsequent round of monthly testing up to November 2020.^15^ A serosurvey of all Norwegian adults (≥20 years)^25^ similarly found greater odds of seropositivity in the first wave for healthcare and transport workers compared with the general population, and in the second wave for food service workers, transport workers and travel stewards, adjusting for age, sex, testing behaviour and maternal country of birth. These temporal changes likely reflect the impact of widespread community transmission and of changing employment and ‘lockdown’ measures.

Few current estimates of differential infection risk across occupations are adjusted for potential sociodemographic confounders, such as deprivation. Furthermore, epidemiological investigation into the mechanisms underlying occupational differences in SARS-CoV-2 infection risk, required to inform evidence-based public health interventions, is lacking. The UK Office for National Statistics (ONS) Coronavirus Infection Survey found little evidence of differential risk of antigen test positivity across occupations (01 September 2020–7 January 2021) after adjusting for a range of sociodemographic factors and the ability to work from home and socially distance at work.^26^ While the study tentatively concluded that contact driven by workplace attendance and ability to socially distance is likely an important driver of occupational differences—and consequently that controlling for these factors suppressed occupational differences—this hypothesis could not be directly tested and disaggregated from the effects of other sociodemographic factors. A prospective serological study of workers from several institutions representing different sectors (healthcare workers, office workers and police) in Milan, Italy up to October 2020 found that workers who continued to travel to their workplace and had daily work-related contact with more than 10 other people and/or direct contact with patients with COVID-19 had elevated risk of seropositivity, after adjustment for age and prior experience of COVID-19 symptoms.^27^ Further investigation into the effect of workplace contact, including a broader range of occupational groups and later phases of the pandemic, is required.

Building on this evidence, we aimed to address gaps in the literature around occupational differences in SARS-CoV-2 infection risk using data from the Virus Watch^28^ community cohort study in England and Wales. Specific research questions were:

1. How do odds of SARS-CoV-2 anti-nucleocapsid seropositivity vary across occupations? (primary objective)
2. Does frequency of work-related close contact mediate the relationship between occupation and seropositivity? (primary objective)
3. How does exposure to poorly ventilated environments vary across occupations? (secondary objective)

## Methods

### Participants

Participants in the current study were a subset of the Virus Watch Study cohort. Virus Watch is a community prospective cohort study of acute respiratory infection syndromes and SARS-CoV-2 infection in England and Wales (n=50 765 as of 08 June 2021). The study includes weekly reporting of symptoms, testing and vaccination status, as well as detailed monthly questionnaires around sociodemographic, health-related and psychosocial/behavioural factors. The eligibility criteria, recruitment strategy, aims and procedures for the Virus Watch Study have been described in detail elsewhere,^28^ with relevant elements for the present study outlined here.

Participants in the present study comprised of adults (≥18 years) who conducted monthly antibody testing in addition to completing the main study surveys. Participants were eligible for inclusion in the present study if:

1. They self-reported their occupation upon study registration.
2. They had a valid antibody test result conducted between 01 February 2021 and 08 June 2021.
3. They responded to the February 2021 monthly survey regarding features of work during the pandemic.

### Exposure

Occupation, the exposure of interest, was semiautomatically coded based on responses to the Virus Watch baseline survey using Cascot V.5.6.3^29 30^ —the UK ONS-recommended occupational coding software—and collapsed into the following categories based on UK Standard Occupational Classification 2020 codes^30 31^: administrative and secretarial occupations; healthcare occupations; indoor trade, process and plant occupations; leisure and personal service occupations; managers, directors, and senior officials; outdoor trade occupations; sales and customer service occupations; social care and community protective services; teaching education and childcare occupations; transport and mobile machine operatives; and other professional and associate occupations (professional and associate professional occupations excluding healthcare, teaching, and social care/community protective services). Please see ‘Occupational Classification’ in the online supplemental materials for further methodological details of exposure classification.

10.1136/oemed-2021-107920.supp1 Supplementary data

### Outcome

The primary outcome was binary serological status (positive vs negative) for SARS-CoV-2 anti-nucleocapsid antibodies—a marker of natural infection. Serological status was determined using self-administered capillary samples which were laboratory tested using the Roche Elecsys anti-N total immunoglobulin assay (cut-off index ≥0.1 indicating seropositivity). Void (invalid) results were excluded. Participants who provided multiple samples were considered seropositive if any sample was above the cut-off value.

The secondary outcome was frequency of workplace exposure to poorly ventilated environments, based on the following question in the February monthly survey: ‘How often do you work indoors in an environment that is never or rarely ventilated (windows or doors opened to let in fresh air or mechanical ventilation system)?’ Responses were classified as Never (never/not applicable), Intermediate (once a month or more—once a week or more but not every day) and Every Day.

### Potential mediator

Frequency of work-related close contact was classified as follows according to participants’ responses to the following question in the February monthly survey, with responses corresponding to their current work situation at the time of the survey: ‘How often does (name/surname)’s work require close contact with others (within 2 m, including with precautions)?’: Never (never/not applicable for example, work from home), Intermediate (once a month or more—once a week or more but not every day) and Every Day. This question was displayed to participants who reported being in full-time or part-time employment or self-employment at the time of the survey.

### Covariates

We identified potential confounders based on a purpose-developed directed acyclic graph (online supplemental figure S2) and the VanderWeele principle of confounder selection.^32^ The following covariates were included to provide a minimally adjusted unbiased estimate of the total and direct effects of occupation, with data drawn from the Virus Watch baseline registration questionnaire: age (<25, 30–39, 40–49, 50–59, 60+ years), sex at birth, minority ethnicity (white British vs other), geographical region (ONS national region) and deprivation based on annual household income (£0–24 900, £25 000–£49 999, £50 000–£75 000 and £75 000+). Based on our directed acyclic graph, the effects of other key sociodemographic confounders—such as ethnicity, household size and underlying health conditions—were addressed through the covariates included in our analyses (see online supplemental figure S2).

### Statistical analyses

For all analyses, ‘other professional and associate’—the most prevalent occupational group (table 1)—was used as the reference category. This group broadly comprised of pre-pandemic office-based/non-frontline occupations (online supplemental table S1) and, following similar selection criteria to previous studies of occupation and COVID-19 outcomes,^5 10 22^ was selected to provide comparison to a large group with plausibly low work-related exposure and absolute infection risk (online supplemental table S2). We investigated the total effect of occupation on serological status and potential mediation by frequency of work-related close contact controlling for age, sex, ethnicity, region and household income (see online supplemental figure S2) using the Buis^33 34^ logistic decomposition method with the *ldecomp* command in Stata V.16.

**Table 1:** Demographic features of participants

Characteristic	Virus Watch full cohort	Current study participants
	N=50 765*	N=3775*
Age		
<30	11 842 (23%)	212 (5.6%)
30–39	5411 (11%)	468 (12%)
40–49	6198 (12%)	782 (21%)
50–59	8186 (16%)	1302 (34%)
60+	19 128 (38%)	1011 (27%)
Sex		
Female	23 427 (46%)	2134 (57%)
Male	18 884 (37%)	1635 (43%)
Unknown	8454 (17%)	6 (0.2%)
Occupation		
Administrative and secretarial	2056 (4.1%)	496 (13%)
Healthcare	1225 (2.4%)	327 (8.7%)
Indoor trades, process and plant	1099 (2.2%)	241 (6.4%)
Leisure and personal service	819 (1.6%)	146 (3.9%)
Managers, directors and senior officials	1352 (2.7%)	319 (8.5%)
Other professional and associate	5403 (10.6%)	1301 (34.0%)
Outdoor trades	326 (0.6%)	85 (2.3%)
Sales and customer service	876 (1.7%)	169 (4.5%)
Social care and community protective services	875 (1.7%)	185 (4.9%)
Teaching, education and childcare	1896 (3.7%)	430 (11%)
Transport and mobile machine	382 (0.8%)	76 (2.0%)
Not in employment (≥16 years)†	14 731 (29.0%)	0 (0.0%)
Child (≤15 years)‡	6548 (12.9%)	0 (0.0%)
Unknown or unable to code	13 177 (26.0%)	0 (0.0%)
Ethnicity		
White British	34 196 (67.4%)	3299 (87%)
White Irish	601 (1.2%)	47 (1.2%)
White Other	2536 (5.0%)	265 (7.0%)
South Asian	2687 (5.3%)	57 (1.5%)
Other Asian	397 (0.8%)	23 (0.6%)
Black	468 (0.9%)	17 (0.5%)
Mixed	891 (1.7%)	44 (1.2%)
Other ethnicity	261 (0.5%)	13 (0.3%)
Unknown	8728 (17.2%)	10 (0.3%)
Household income		
£0–£24 999	9907 (19.5%)	525 (14%)
£25 000–£49 999	11 893 (23.4%)	1159 (31%)
£50 000–£74 999	7271 (14.3%)	899 (24%)
£75 000+	7790 (15.3%)	968 (26%)
Unknown	13 904 (27.4%)	219 (5.8%)
Region		
East Midlands	4183 (8.2%)	327 (8.7%)
East of England	9433 (19%)	865 (23%)
London	8444 (17%)	560 (15%)
North East	2218 (4.4%)	158 (4.2%)
North West	4670 (9.2%)	411 (11%)
South East	8346 (16%)	767 (20%)
South West	3141 (6.2%)	251 (6.6%)
Wales	1043 (2.1%)	68 (1.8%)
West Midlands	2350 (4.6%)	188 (5.0%)
Yorkshire and The Humber	2483 (4.9%)	180 (4.8%)
Unknown	4454 (8.8%)	0 (0.0%)

*n (%).

**Figure 1:**
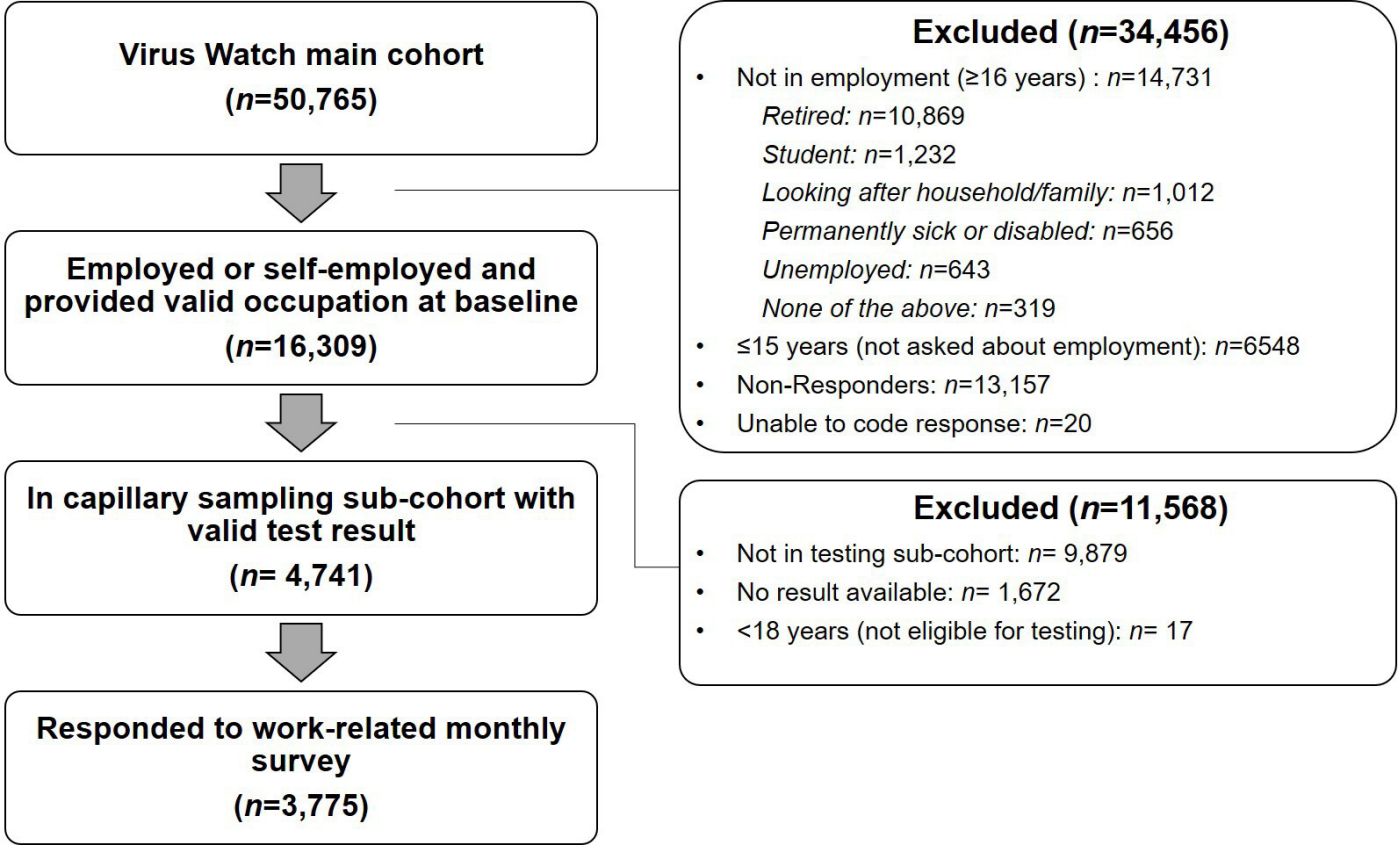
Flow diagram of participant eligibility.

†See figure 1 for further detail.

‡Not asked about employment.

We investigated the relationship between occupation and frequency of exposure to poorly ventilated workplaces using ordered logistic regression. This model was not adjusted for sociodemographic factors as a relationship between these factors and exposure to poorly ventilated workspaces was assumed to occur due to occupation. While poor workplace ventilation is a plausible moderator of the indirect effect of workplace contact, it was not included in a moderated-mediation model as it was not possible to determine if the close contacts reported also occurred in poorly ventilated spaces and lack of statistical power.

There were no missing data for occupation, workplace contact frequency, age or national region. Minimal data were missing for workplace exposure to poor ventilation (0.6%, n=24), sex (0.2%, n=6) and ethnicity (0.3%, n=10). Household income was missing for 5.8% of participants (n=219); available data were entered into models. We conducted a sensitivity analysis after performing multivariate imputation by chained equations (*mice* package in R V.4.0.3) with 5 datasets with 50 iterations per dataset to sociodemographic covariates for the mediation models.

## Results

Selection of participants for inclusion in the present study is illustrated in figure 1. Table 1 reports demographic features of the full Virus Watch cohort (n=50 765) and of participants included in the present study (n=3775).

### Total effect of occupation on SARS-CoV-2 serological status

The proportion of seropositive and seronegative participants by occupation is reported in online supplemental table S2. Logistic regression adjusted for age, sex, ethnicity, household income and national region (table 2 (total effect)) found that participants employed in healthcare professions (OR=2.46, 95% CI 1.82 to 3.33), indoor trade, process and plant occupations (OR=2.07, 95% CI 1.38 to 3.12), leisure and personal service occupations (OR=1.23, 95% CI 1.03 to 3.14), and transport and mobile machine operatives (OR=2.17, 95% CI 1.04 to 4.50) had greater total odds of SARS-CoV-2 seropositivity compared with participants in the ‘other professional and associate’ category.

**Table 2:** ORs for total, indirect and direct effects

	Total*			Indirect*			Direct*		
	OR	95% CI	P value	OR	95% CI	P value	OR	95% CI	P value
Other professional and associate	Ref	Ref	Ref	Ref	Ref	Ref	Ref	Ref	Ref
Administrative and secretarial	1.29	0.89 to 1.85	0.18	1.04	1.01 to 1.08	0.01	1.23	0.85 to 1.78	0.27
Healthcare	2.46	1.82 to 3.33	<0.001	1.23	1.09 to 1.40	0.001	2.00	1.43 to 2.79	<0.001
Indoor trades, process and plant	2.07	1.38 to 3.12	<0.001	1.17	1.06 to 1.30	0.003	1.77	1.16 to 2.69	0.01
Leisure and personal service	1.80	1.03 to 3.14	0.04	1.14	1.04 to 1.25	0.01	1.58	0.92 to 2.74	0.10
Managers, directors and senior officials	1.17	0.78 to 1.77	0.45	1.04	1.003 to 1.08	0.03	1.13	0.75 to 1.69	0.56
Outdoor trades	1.61	0.83 to 3.10	0.16	1.13	1.04 to 1.23	0.005	1.42	0.74 to 2.75	0.29
Sales and customer service	1.53	0.87 to 2.67	0.14	1.11	1.04 to 1.18	0.002	1.38	0.78 to 2.45	0.27
Social care and community protective services	1.41	0.86 to 2.32	0.18	1.12	1.04 to 1.21	0.005	1.26	0.75 to 2.12	0.38
Teaching, education and childcare	1.17	0.85 to 1.61	0.33	1.12	1.04 to 1.21	0.002	1.04	0.75 to 1.46	0.80
Transport and mobile machine	2.17	1.04 to 4.50	0.04	1.23	1.08 to 1.40	0.002	1.77	0.87 to 3.61	0.12

*Total effect=the effect of occupation prior to adjustment for the mediator (work-related close contact); indirect effect=the effect of occupation on odds of seropositivity mediated through work-related close contact; direct effect=the effect of occupation excluding mediation by work-related close contact.

### Mediation analysis for workplace contact frequency

Workplace contact frequency by occupation is reported in online supplemental table S3. Anti-nucleocapsid seropositivity was 17.1% (123 of 721) among workers with daily close contact, compared with 13.2% (125 of 950) for those with intermediate-frequency contact and 10.0% (210 of 2104) for those who worked from home or never had close contact with others at work (online supplemental table S3). Results of the models for the indirect and the direct effects are reported in table 2. There were positive indirect effects (ie, OR >1.00) with bootstrapped CIs that excluded the value one across occupational groups, suggesting mediation of the occupation–seropositivity relationship by work-related contact frequency (OR range 1.04 (95% CI 1.003 to 1.08) − 1.23 (95% CI 1.09 to 1.40)). After accounting for the indirect effect of workplace contact frequency, a positive direct effect of occupation on serological status remained for healthcare professions (OR 2.00, 95% CI 1.43 to 2.79), and indoor trade, process and plant occupations (OR 1.77, 95% CI 1.16 to 2.69) (table 2). Consistent results were obtained in the sensitivity analysis with missing sociodemographic data imputed (online supplemental table S4).

### Exposure to poorly ventilated environments by occupation

**Figure 2:**
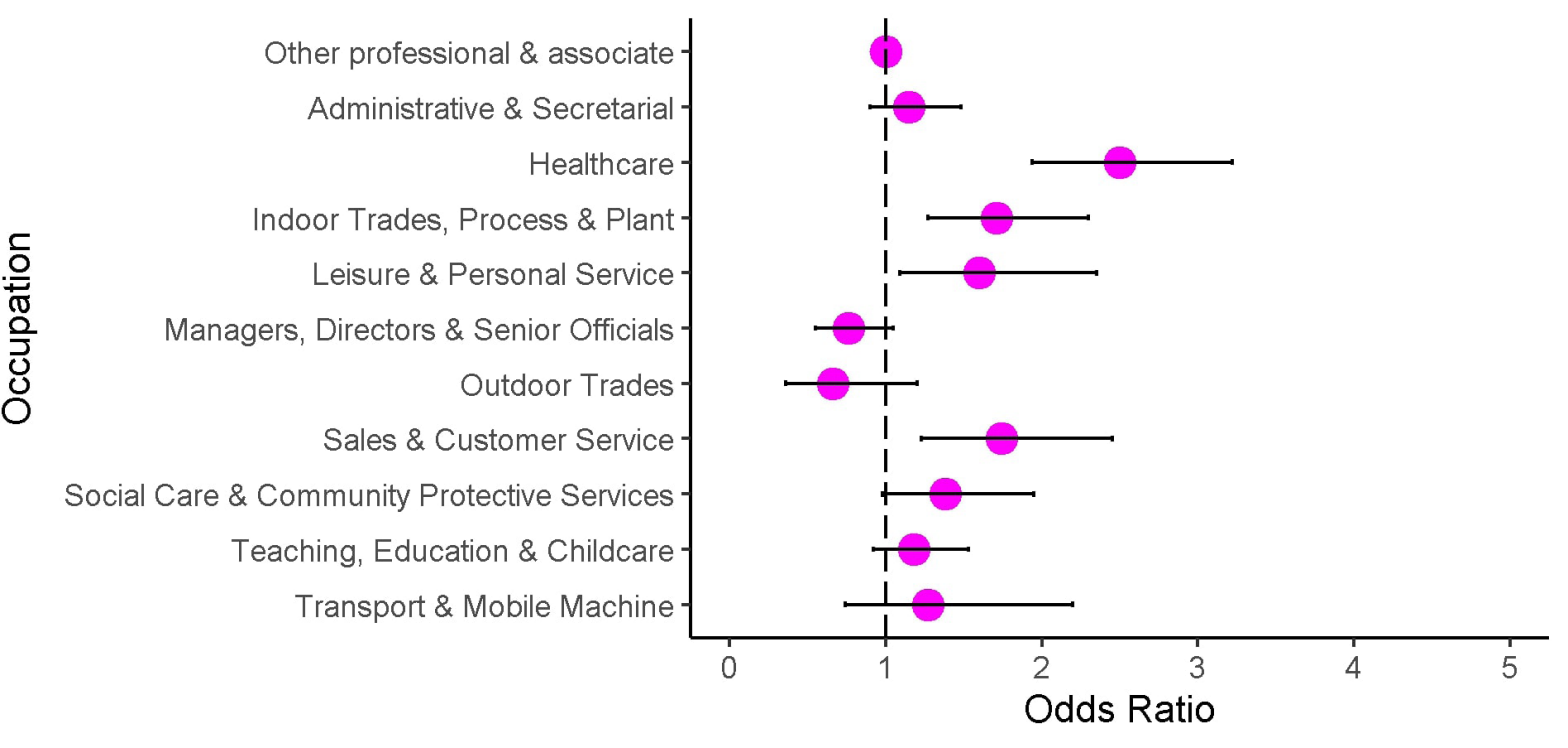
Crude ORs for frequency of exposure to poorly ventilated workplace by occupation.

Frequency of exposure to poorly ventilated environments is reported in online supplemental table S5. Anti-nucleocapsid seropositivity was 18.4% (75 of 408) among those who had daily exposure to poorly ventilated workplaces, compared with 13.2% (67 of 508) of those with intermediate exposure and 11.0% (312 of 2835) of those who never had exposure to poorly ventilated workplaces (further detail in online supplemental table S5). Ordered logistic regression (figure 2 and online supplemental table S6) indicated that—compared with participants in the ‘other professional and associate’ category—participants employed in healthcare professions (OR=2.50, 95% CI 1.94 to 3.22), indoor trade, process and plant occupations (OR=1.71, 95% CI 1.27 to 2.30), leisure and personal service occupations (OR=1.60, 95% CI 1.09 to 2.35), and sales and customer service occupations (OR=1.74, 95% CI 1.23 to 2.45) had elevated crude odds of more frequent exposure to poorly ventilated workplace environments.

## Discussion

### Main findings of this study

Findings from this prospective cohort study in England and Wales indicated that healthcare workers, indoor trade, process and plant workers, leisure and personal service workers, and transport and mobile machine operatives had around twice the total odds of seropositivity compared with participants in other professional and associate occupations, adjusted for age, sex, ethnicity, household income and national region. Anti-nucleocapsid seropositivity was highest among those with daily close contact at work (17%) and lowest in those who never had work-related close contact (10%). Frequency of work-related close contact explained a variable but substantial part of the increased odds of infection in high-risk occupational groups. After accounting for workplace close contact, healthcare workers and indoor trade, process and plant workers had residual increased odds of infection, suggesting that other work-related factors also contribute to their increased risk.^35^ Healthcare workers and indoor trade, process and plant workers also had greater odds of reporting frequent exposure to poor indoor ventilation at work. Mediation models based on observational data must be interpreted with caution in relation to causal inference (see further discussion in online supplemental materials). Nevertheless, the finding that workplace contact frequency explains a considerable part of the variation in occupational risk is biologically plausible given the transmission routes of SARS-CoV-2.^36 37^

### Strengths and limitations

Key strengths of this work include objective measurement of prior infection status through nucleocapsid antibody testing, which should be unaffected by vaccination. This provides a cumulative measure of infection risk through the first and second waves of the pandemic in England and Wales. Furthermore, through use of a mediation model informed by a directed acyclic graph, we were able to investigate a putative mechanism for increased occupational risk—work-related close contact. Finally, we were able to investigate variation in exposure to poorly ventilated workplace settings across occupational groups.

Key limitations include that timing of infection cannot be ascertained, and that antibody waning may lead to false negative results.^13^ Frequency of work-related contact was measured during the second wave and may have changed over time. However, legislation and guidance around workplace closures were broadly similar for many occupations across periods with the highest levels of SARS-CoV-2 transmission. Work-related contact frequency was self-reported in broad categories and could not fully account for risk-relevant features of contact—such as the number and duration of close contacts or the presence of risk-mitigating methods such as PPE—or distinguish those who worked from home from those who attend workplaces but never have close contact. Notably, differential access to effective PPE at work plausibly moderates the effect of direct and indirect contact on infection risk, and is an important area for further investigation. We were also unable to assign a source (eg, colleagues and/or the public) of close contact at work, and this is recommended for future work in order to inform sector-specific interventions.

Relatively small sizes of some occupational groups likely impacted the precision of estimates. Some covariates required broad categorisation to retain statistical power, and the complex inter-relationships and challenges measuring sociodemographic confounders make excluding confounding effects difficult. We did not control for vaccination, although many working-age adults—excluding healthcare workers—would be unvaccinated during the study period. Further, confounding by occupation-related non-workplace contact, for example, using public transport to reach work, is plausible. Nevertheless, our findings suggest that work-related close contact is an important explanatory variable for differential infection risk across occupational groups.

Reporting of workplace contact frequency and ventilation may have been affected by recall bias. We lacked appropriately detailed measurement and power to include ventilation within the mediation model. Detailed features and effectiveness of mechanical ventilation are likely to be difficult for a non-specialist to assess. We were not able to directly explore what accounted for the residual risk in healthcare workers and indoor trade and process and plant workers.

Finally, it should be noted that this analysis covers periods of intense restrictions. Occupational patterns are likely to change considerably as restrictions are lifted.

### What is already known on this topic and what this study adds

The elevated total odds of seropositivity identified in the present study among healthcare workers, indoor trade, process and plant occupations, leisure and personal service occupations, and transportation occupations broadly corroborate previous findings in the UK and worldwide indicating elevated risk of infection^19 21–25^ in similar groups. These findings support and build on the important role of work-related contact suggested in the ONS Coronavirus Infection Survey.^26^ Differential infection risk influenced by work-related contact could plausibly contribute to variations in occupational morbidity and mortality observed in other studies.^5–9 12^ Working from home may drastically reduce this occupational risk and contribute to reducing infections. However, differential ability to work from home may exacerbate occupational and social inequalities. The extent to which work from home should continue to be encouraged as other restrictions are lifted is an important consideration for society globally. Reducing footfall and maintaining social distancing in the workplace may also be important. The relative importance of these measures will depend on infection levels, vaccination levels and the effectiveness of vaccines against current and future variants of SARS-CoV-2.

High risk in healthcare workers is well described previously,^13–20^ though accounting for variation between specific occupations and over time due to changing PPE provision and infection control practices was beyond the scope of this study. Measures to improve ventilation are likely to be important for control of nosocomial transmission of SARS-CoV-2 and other respiratory infections. Residual risk in indoor trade and process and plant workers, combined with greater self-reported exposure to poor ventilation, also represents an important area for further investigation and modification to reduce risk. The extent and effectiveness of ventilation is likely to vary considerably according to the design of such workplaces.

## Conclusion

This study was able to compare occupational risk of SARS-CoV-2 infection after controlling for a range of sociodemographic confounders, and indicates that occupation has an important independent association with SARS-CoV-2 infection. Frequency of close contact at work is suggested to explain a considerable amount of this variation. Reducing work-related close contact through measures such as social distancing and working from home is likely to have played an important role in controlling COVID-19 transmission. Poor ventilation in some workplace settings may also contribute to risk, and presents an important area for further inquiry.

## Data Availability

No data are available. We aim to share aggregate data from this project on our website and via a 'Findings so far' section on our website (https://ucl-virus-watch.net/). We will also be sharing individual record level data on a research data sharing service such as the Office for National Statistics Secure Research Service. In sharing the data, we will work within the principles set out in the UKRI guidance on best practice in the management of research data. Access to use of the data while research is being conducted will be managed by the chief investigators (AH and RA) in accordance with the principles set out in the UKRI guidance on best practice in the management of research data. We will put analysis code on publicly available repositories to enable their reuse.
