# Supplementary material for: Occupation, work-related contact and SARS-CoV-2 anti-nucleocapsid serological status: findings from the Virus Watch prospective cohort study
Source: Occup Environ Med. 2022 Apr 21;79(11):729–35. doi: 10.1136/oemed-2021-107920 (PMC9072780; doi:10.1136/oemed-2021-107920)
Supplement: Supplementary data [file oemed-2021-107920supp001.pdf]

Supplementary Materials

Supplementary Figure S1. Total Effect and Mediation Models

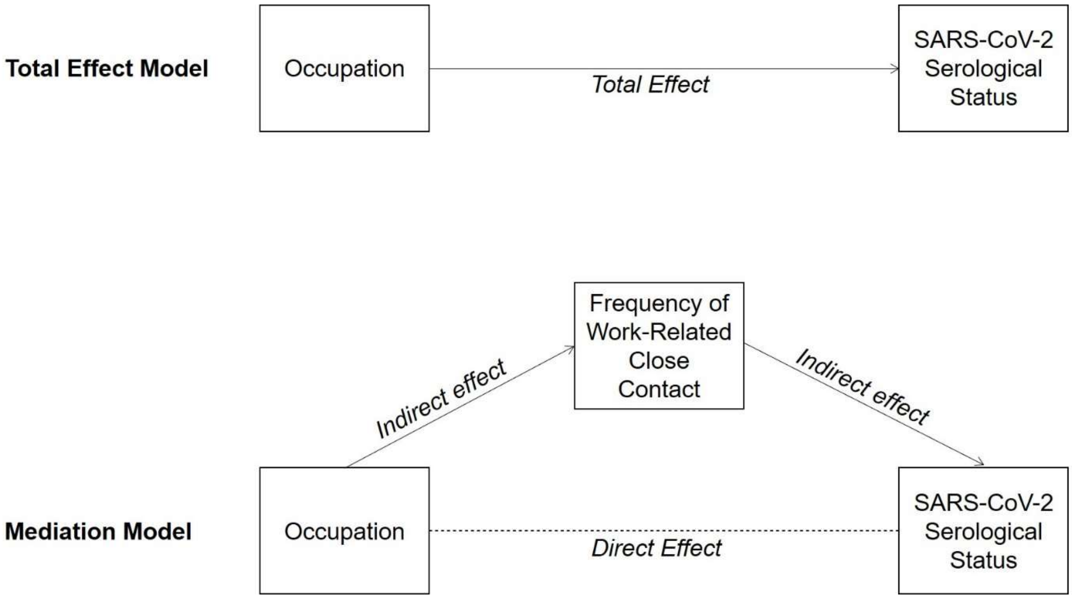

Discussion of Mediation Models

Total-effect models differ fundamentally from mediation models, and the absence of a total effect does not necessarily indicate the absence of theoretically-sound mediated effects, though these should be interpreted cautiously [1,2]. Following Zhao et al.'s typology of mediation effects [1], our findings suggested indirect-only mediation for all occupational groups except for healthcare workers and indoor trade, process and plant occupations. Direct effects were observed in the mediation model for the latter groups, consistent with complementary mediation[36] and suggesting the presence of further indirect effects unaccounted for in the model.

1 Zhao X, Lynch JG, Chen Q. Reconsidering Baron and Kenny: Myths and Truths about Mediation Analysis. *J Consum Res* 2010; **37**: 197–206.

2 Preacher KJ. Advances in mediation analysis: a survey and synthesis of new developments. *Annu Rev Psychol* 2015; **66**: 825–52.

Supplementary Figure S2. Directed Acyclic Graph for Estimating Total and Contact-Mediated Effect of Occupation on SARS-CoV-2 Infection Risk

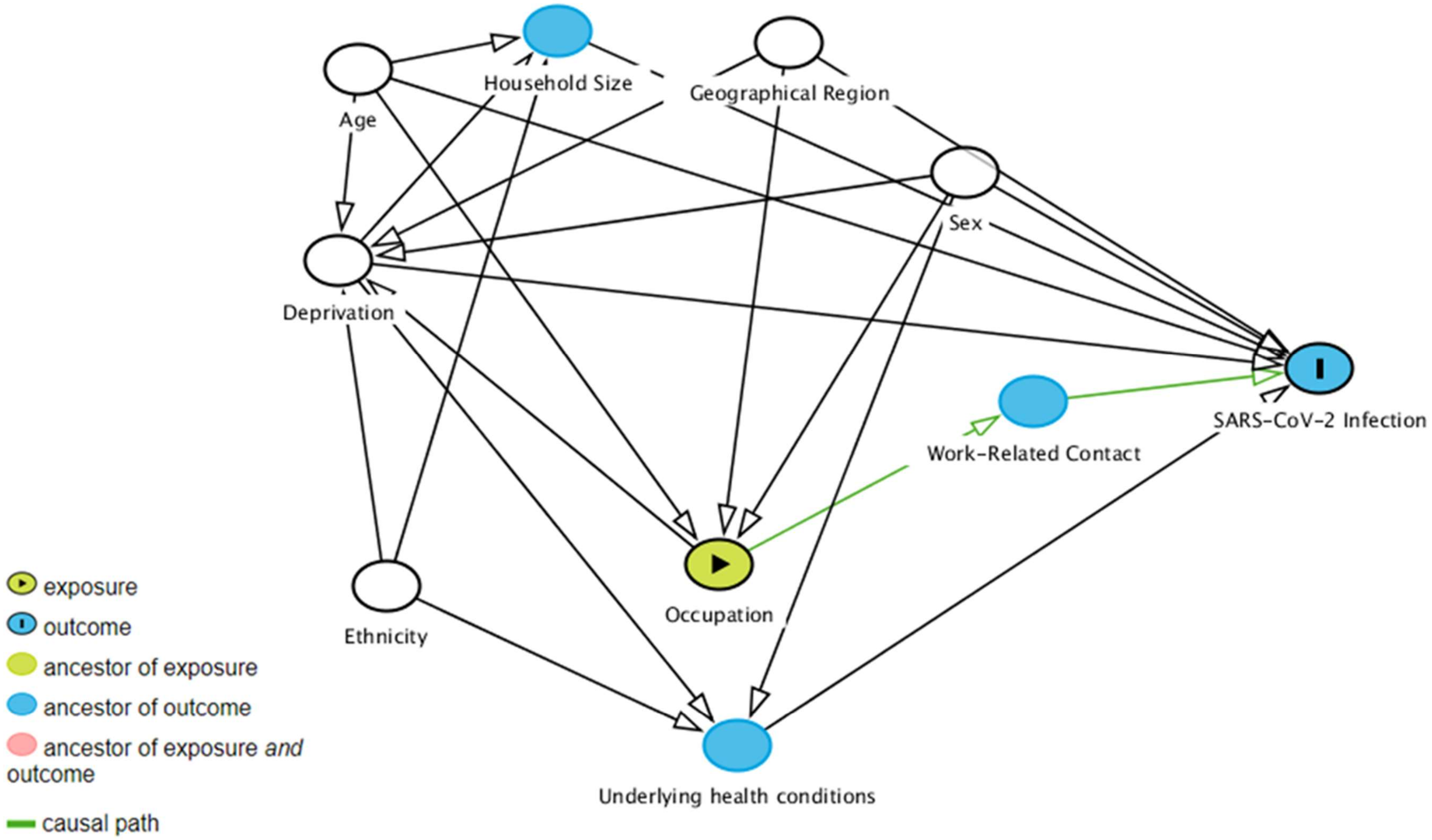

Occupational Classification

UK Standard Occupational Classification 2020 (SOC-2020) codes are grouped within minor, sub-major, and major categories based on occupation-related skill level and skill specialisation. As these groupings combine occupations that take place in varying environments and with different work-related behaviour/public exposure patterns, we categorised SOC-2020 codes into the following occupational categories to reflect these factors while retaining, where possible, the occupational groups outlined by ONS: administrative and secretarial occupations; healthcare occupations; indoor trade, process & plant occupations; leisure and personal service occupations; managers, directors, and senior officials; outdoor trade occupations; sales and customer service occupations; social care and community protective services; teaching education and childcare occupations; transport and mobile machine operatives; and other professional and associate occupations (professional and associate professional occupations excluding healthcare, teaching, and social care/community protective services).

Supplementary Table S1 lists UK 2020 SOC codes included within each occupational category, and the most prevalent SOC-2020-defined occupations within each category.

Supplementary Table S1. UK Standard Occupational Classification 2020 (SOC-2020) Codes within Virus Watch Occupational Categories

Virus Watch Occupational Category	UK SOC-2020 Codes	Three Most Prevalent Occupations* (SOC-2020 Unit Group)
Administrative & Secretarial Occupations	4111-4217, 9211, 9219, 9233	1. Other administrative occupations n.e.c. (25%, n= 122)<br>2. Book-keepers, payroll managers, and wage clerks (11%, n=55)<br>3. Personal assistants and other secretaries (7%, n=34)
Healthcare Occupations	2211-2259, 3211-3219, 3240, 6131-6133	1. Other nursing professionals (22%, n=73)<br>2. Generalist medical practitioners (10%, n=33)<br>3. Nursing auxiliaries and assistants (7%, n=24)
Indoor Trades, Process & Plant Occupations	5211-5250, 5315-5317, 5321-5323, 5411-5449, 8111-8149, 8160, 9131-9139, 9241-9259	1. Warehouse operatives (12%, n=28)<br>2. Metalworking production and maintenance fitters (10%, n=23)

		3. Electricians and electrical fitters (8%, n=20)
Leisure & Personal Service Occupations	1221-1225, 1252, 1253, 1256, 1257, 6121, 6129, 6211-6250, 9221-9229, 9231, 9261-9269	1. Cleaners and domestics (14%, n=21)<br>2. Hairdressers and barbers (10%, n=15)<br>3. Animal care service occupations n.e.c. (7%, n=10)
Managers, Directors & Senior Officials	1111-1161, 1171,1172, 1211, 1212, 1231, 1241-1243, 1251, 1254, 1255, 1258, 1259	1. Financial managers and directors (18%, n=59)<br>2. Functional managers and directors n.e.c. (10%, n=31)<br>3. Human resource managers and directors (9%, n=30)
Other Professionals & Associate Professionals	2111-2162, 2411-2455, 2471-2494, 3111-3133, 3411-3582	1. Programmers and software development professionals (6%, n=78)<br>2. Management consultants and business analysts (4%, n=56)<br>3. Business and financial project management professionals (4%, n=52)
Outdoor Trade Occupations	5111-5119, 5311-5314, 5319, 5330, 8151-8159, 9111- 9129	1. Gardeners and landscape gardeners (20%, n=17)<br>2. Farmers (18%, n=15)<br>3. Construction and building trades n.e.c. (14%, n=12)
Sales & Customer Service Occupations	7111-7220	1. Sales and retail assistants (37%, n=62)<br>2. Sales supervisors - retail and wholesale (12%, n=20)<br>3. Customer service occupations n.e.c. (10%, n=17)

Social Care & Community Protective Services	1162, 1163, 1232, 2461-2469, 3221-3229, 3311-3319, 6134-6138, 6311-6312	1. Care workers and home carers (23%, n=43)<br>2. Welfare and housing associate professionals n.e.c. (12%, n=22)<br>3. Social workers (9%, n=16)
Teaching, Education & Childcare Occupations	1233, 2311-2329, 3231, 3232, 6111-6117, 9232	1. Secondary education teaching professionals (14%, n=59)<br>2. Higher education teaching professionals (13%, n=58)<br>3. Education advisers and school inspectors (13%, n=56)
Transport & Mobile Machine Operatives	8211-8239	1. Large goods vehicle drivers (22%, n=17)<br>2. Driving instructors (14%, n=11)<br>3. Bus and coach drivers (13%, n=10)

Abbreviations: n.e.c. = not elsewhere classified; * Limited to three most prevalent occupations per category to prevent declarative disclosure and due to large number of occupations across sample (*n*=371)

Supplementary Table S2. SARS-CoV-2 Serological Status by Occupation

Characteristic	Negative, N = 3,317[1]	Positive, N = 458[1]
Occupation		
Administrative & Secretarial	441.0 (88.9%)	55.0 (11.1%)
Healthcare	262.0 (80.1%)	65.0 (19.9%)
Indoor Trades, Process & Plant	203.0 (84.2%)	38.0 (15.8%)
Leisure & Personal Service	122.0 (83.6%)	24.0 (16.4%)
Managers, Directors & Senior Officials	286.0 (89.7%)	33.0 (10.3%)
Other professional & associate	1,173.0 (90.2%)	128.0 (9.8%)
Outdoor Trades	73.0 (85.9%)	12.0 (14.1%)
Sales & Customer Service	148.0 (87.6%)	21.0 (12.4%)
Social Care & Community Protective Services	163.0 (88.1%)	22.0 (11.9%)
Teaching, Education & Childcare	382.0 (88.8%)	48.0 (11.2%)
Transport & Mobile Machine	64.0 (84.2%)	12.0 (15.8%)

[1]n (row %)

Supplementary Table S3. Frequency of Workplace Close Contact by Seropositivity and Occupation

Characteristic		Daily, N = 721[1]	Intermediate, N = 950[1]	Never, N = 2,104[1]
Occupation	Other professional & associate	85.0 (6.5%)	242.0 (18.6%)	974.0 (74.9%)
	Administrative & Secretarial	64.0 (12.9%)	125.0 (25.2%)	307.0 (61.9%)
	Healthcare	151.0 (46.2%)	106.0 (32.4%)	70.0 (21.4%)
	Indoor Trades, Process & Plant	82.0 (34.0%)	82.0 (34.0%)	77.0 (32.0%)
	Leisure & Personal Service	40.0 (27.4%)	45.0 (30.8%)	61.0 (41.8%)
	Managers, Directors & Senior Officials	45.0 (14.1%)	66.0 (20.7%)	208.0 (65.2%)
	Outdoor Trades	20.0 (23.5%)	34.0 (40.0%)	31.0 (36.5%)
	Sales & Customer Service	38.0 (22.5%)	51.0 (30.2%)	80.0 (47.3%)
	Social Care & Community Protective Services	43.0 (23.2%)	66.0 (35.7%)	76.0 (41.1%)
	Teaching, Education & Childcare	119.0 (27.7%)	112.0 (26.0%)	199.0 (46.3%)
	Transport & Mobile Machine	34.0 (44.7%)	21.0 (27.6%)	21.0 (27.6%)
Anti-Nucleocapsid Seropositive	Yes	123 (17.1%)	125 (13.2%)	210 (10.0%)
	No	598 (82.9%)	825 (86.8%)	1894 (90.0%)

[1]n (%)

Supplementary Table S4. Odds Ratios for Total, Indirect, and Direct Effects

	Total			Indirect			Direct		
	**OR**	**95% CI**	***p***	**OR**	**95% CI**	***p***	**OR**	**95% CI**	***p***
Other Professional & Associate	REF	REF	REF	REF	REF	REF	REF	REF	REF
Administrative & Secretarial	1.29	0.89, 1.85	0.18	1.04	1.01, 1.08	0.02	1.23	0.86, 1.76	0.25
Healthcare	2.46	1.80, 3.37	<0.001	1.23	1.08, 1.41	0.002	2.00	1.47, 2.72	<0.001
Indoor Trades, Process & Plant	2.07	1.40, 3.07	<0.001	1.17	1.05, 1.31	0.004	1.77	1.21, 2.59	0.003
Leisure & Personal Service	1.80	1.12, 2.90	0.02	1.14	1.03, 1.25	0.01	1.58	1.02, 2.46	0.04
Managers, Directors & Senior Officials	1.17	0.74, 1.85	0.50	1.04	1.00, 1.08	0.03	1.13	0.72, 1.77	0.61
Outdoor Trades	1.61	0.72, 3.56	0.24	1.13	1.03, 1.23	0.01	1.42	0.65, 3.13	0.38
Sales & Customer Service	1.53	0.86, 2.70	0.15	1.11	1.03, 1.19	0.01	1.38	0.78, 2.45	0.27
Social Care & Community Protective Services	1.41	0.87, 2.29	0.17	1.12	1.04, 1.21	0.004	1.26	0.78, 2.04	0.35
Teaching, Education & Childcare	1.17	0.88, 1.57	0.29	1.12	1.03, 1.23	0.01	1.04	0.78, 1.40	0.78
Transport & Mobile Machine	2.17	1.18, 3.99	0.01	1.23	1.06, 1.42	0.01	1.77	0.96, 3.26	0.07

Supplementary Table S5. Frequency of Exposure to Poorly Ventilated Workplace by Occupation

Characteristic		**Daily, N = 408[1]**	**Intermediate, N = 508[1]**	**Never, N = 2,835[1]**
	Other professional & associate	128.0 (9.9%)	138.0 (10.7%)	1,029.0 (79.5%)
	Administrative & Secretarial	55.0 (11.2%)	58.0 (11.8%)	380.0 (77.1%)
	Healthcare	57.0 (17.6%)	74.0 (22.9%)	192.0 (59.4%)
	Indoor Trades, Process & Plant	30.0 (12.6%)	45.0 (18.9%)	163.0 (68.5%)
	Leisure & Personal Service	23.0 (16.1%)	18.0 (12.6%)	102.0 (71.3%)
	Managers, Directors & Senior Officials	25.0 (7.9%)	27.0 (8.5%)	265.0 (83.6%)
	Outdoor Trades	2.0 (2.4%)	11.0 (12.9%)	72.0 (84.7%)
	Sales & Customer Service	23.0 (13.7%)	30.0 (17.9%)	115.0 (68.5%)
	Social Care & Community Protective Services	19.0 (10.3%)	31.0 (16.8%)	135.0 (73.0%)
	Teaching, Education & Childcare	35.0 (8.2%)	69.0 (16.1%)	325.0 (75.8%)
	Transport & Mobile Machine	11.0 (14.7%)	7.0 (9.3%)	57.0 (76.0%)
Anti-Nucleocapsid Seropositive	Yes	75 (18.4%)	67 (13.2%)	312 (11.0%)
	No	333 (81.6%)	441 (86.8%)	2523 (89.0%)

[1]n (%)

Supplementary Table S6. Odds Ratios for Frequency of Exposure to Poorly Ventilated Workplace by Occupation

	OR	95% CI	*p*
Other professional & associate	REF	REF	REF
Administrative & Secretarial	1.15	0.90,1.48	0.26
Healthcare	2.50	1.94,3.22	<0.001
Indoor Trades, Process & Plant	1.71	1.27,2.30	<0.001
Leisure & Personal Service	1.60	1.09,2.35	0.02
Managers, Directors & Senior Officials	0.76	0.55,1.05	0.10
Outdoor Trades	0.66	0.36,1.20	0.17
Sales & Customer Service	1.74	1.23,2.45	0.002
Social Care & Community Protective Services	1.38	0.98,1.95	0.07
Teaching, Education & Childcare	1.18	0.92,1.53	0.20
Transport & Mobile Machine	1.27	0.74,2.20	0.39
